# On the Sensibility of the Teeth

**Published:** 1850-10

**Authors:** Alfred A. Blandy


### ARTICLE V.

*On the Sensibility of the Teeth.* By ALFRED A. BLANDY, M. D.

Every practitioner of dental surgery has observed the differences in the sensibility of the teeth of different individuals, and even in the same mouth. It is well known that all teeth are not affected alike by external impressions. The application of heat and cold to the teeth of some persons is productive of severe pain, and the same may be said of acids; while upon others, it is attended with but little or no unpleasant sensation. The removal of caries from the teeth of some is attended with insupportable pain, while from others, it is not productive of the slightest suffering. The bone of some teeth, when exposed by the destruction of the enamel or recession of the gums is so very sensitive that the slightest contact of any hard foreign substance causes the most acute pain, while that of others does not seem to be endowed with any perceptible sensibility.

To what are these differences in the sensibility of different teeth attributable? Are they dependant upon constitutional idiosyncrasy, or upon the physical condition of the teeth themselves, or upon some peculiar pathological state of these organs? If of no practical importance, it would at least be interesting to have these questions satisfactorily answered. But it is not so much the purpose of the writer to do this, as it is to elicit the views of older and more experienced practitioners.

In the American Journal of the Medical Sciences of the present month, (October,) there is a short article copied from the Transactions of the College of Physicians of Philadelphia, vol. iii, No. 2, in which Dr. John Neill attempts to explain "the manner in which external impressions made upon the teeth are conveyed to the nervous pulp within." His explanation is founded upon the assumption that the teeth are not endowed with blood-vessels and nerves, and that dentine consists of tubules—an assumption, we think, altogether gratuitous, and by no means in accordance with the present state of our knowlege of the anatomical structure of these organs. Dr. Neill, also, takes it for granted that the old doctrine of increased sensibility of the teeth is the result of inflammation, is exploded—a position, we think, equally unwarranted and wholly at variance with the daily observation of every dentist.

After disposing, in this summary way, of the doctrine of the vascularity and inflammation of tooth-bone, or dentine, he states that the hollow tubules, which pass from the circumference to the center of this structure are filled with a fluid secreted by the pulp. "Now," asks Dr. Neill, "if the tubules thus filled pass from the circumference to the interior of the teeth, may not pressure applied without, by compressing the enamel and the fluid of the tubules, affect the nervous pulp within, by subjecting the latter to a species of hydrostatic pressure, the amount of which can be measured?" He then goes on to say that, "whatever reduces the thickness of the enamel or uncovers any portion of the dentine, increases the painful impressions caused by external pressure. Thus acid, by removing the mucus by which teeth are ordinarily covered and perhaps a portion of the enamel, increases their sensibility to external impressions."

This, it must be confessed, is a most beautiful and ingenious explanation, but, unfortunately, we fear it will not stand the test of rigid scrutiny. If Dr. Neill's assumptions were all correct, however much his theory might be opposed to daily observation, it would, at least, carry with it a considerable amount of plausibility. But let us examine them, for a moment, in detail.

The first is, that "the old doctrine of increased sensibility of the teeth from inflammation has passed away." This, so far from being correct, is, we believe, held by nearly every dentist. The *hydrostatic pressure* doctrine of Dr. Neill, would, in the opinion of the writer wholly fail to explain the differences in the sensibility of different teeth, and even in different parts of the same tooth, apparently in the same pathological state, or, at least, so far as indicated by the physical condition and appearance of the organs and parts. For example, partially decomposed dentine, and even the surrounding sound bone are so exquisitely sensitive that the slightest touch of an excavator or file is productive of the severest pain; at other times, they are so totally devoid of sensibility that they can be cut away without causing the slightest unpleasant feeling to the patient. This difference is sometimes observed in different parts of the same tooth. Now, if the impressions made with the excavator or file, so compressed the fluid in the *supposed tubules* as to subject the pulp within "to a *species of hydrostatic pressure*, causing pain in one case, would it not be likely to do it in all the teeth to which this instrument was applied? Most certainly it would.

The same difference is observed in the susceptibility of different teeth, and even in the same teeth at different times, to impressions from heat and cold and acids. But if this were not the case, and if all teeth, and at all times, were equally subject to impressions from these agents, it is difficult to conceive how the hydrostatic pressure theory of Dr. Neill would explain the manner in which they are made, unless it were by the expansion and contraction of the fluid contained in the tubules of the dentine, compressing the nervous pulp in the one instance, and causing it to expand in the other, to fill the partial vacuum formed in each tubule. But as this is altogether a gratuitous supposition, it seems to the writer, that until a more philosophical explanation is furnished, the nerves of the dentine must continue to be regarded as the media through which impressions made upon the teeth are transmitted to the pulp within. Admitting this doctrine to be true, it is easy to explain the cause of the difference in the susceptibility of different teeth, and in the same teeth at different times, to external impressions—depending as it necessarily does, upon the state of their nervous susceptibility at the time they are made. This is always increased by inflammation of the dentine, and it is also evidently influenced both by the physical condition of the organs and the state of the general system, often manifesting itself when the former are apparently free from increased vascular action. It is also often increased in a most wonderful degree by the action of acids, and especially after the dentine has become exposed by the destruction of the enamel or recession of the gums. There seems, however, to be some difference of opinion with regard to the manner in which this effect is produced. Some ascribe it to increased sensibility in the gums and alveolo-dental periosteum, and not to any direct action of the acid upon the teeth themselves. This opinion, however, is not held by many dentists of the present day, for it is well known that no increase of nervous susceptibility in these organs is produced unless the acid come in direct contact with them. The most probable, and in the opinion of the writer, the only way by which this effect can be produced, is, by the action or union of the acid with the lime of the teeth, decomposing it upon the surface, and leaving the animal frame-work, which is the only part endowed with sensibility, exposed. The sensibility, too, of this exposed animal tissue is, no doubt, increased both by the action of the acid and its exposure.

Teeth are less easily affected by acids previous to the destruction of any portion of the enamel than afterwards, and for the reason, that this outer covering contains much less animal matter than the subjacent dentine; consequently it is endowed with less nervous sensibility. Teeth, too, after the dentine has become exposed, are more susceptible to impressions from heat and cold, and for the same reason. Hence, teeth that are partially worn away, or that have been filed, or filled with a metallic substance, metals being better conductors of caloric than tooth-bone, are more easily affected by these agents.

Acidity of the fluids of the mouth or stomach, is attended by increased sensibility of the teeth; consequently, any state or condition of the general system which favors the former, may be regarded as conducive of the latter. Hence the teeth of dyspeptic persons, and females during gestation, are peculiarly liable to be sensitive. The same, too, as a general rule, may be said of persons possessed of a high degree of constitutional nervous susceptibility—the teeth partaking in a measure of the condition of the general system.

The second assumption of Dr. Neill is, that "the teeth have neither blood-vessels nor nerves." As being in more immediate connection with what has been just said, it may be well to offer a remark on the last part of this proposition, before noticing the first.

Although anatomists have failed to detect nerves in dentine, with the best microscopes, their existence is inferred from the manifestation of phenomena which could not occur in a structure devoid of these organs.

With regard to the vascularity of the osseous portion of a tooth, the writer does not think that that admits of a single doubt.

The existence of vessels here has been fully demonstrated by Drs. Harris, Maynard, Tomes and others.* It is true, the vessels of dentine are so exceedingly minute, that they are only capable of conveying the serous part of blood, though red blood has been detected in some of them in several instances. Dr. Harris has given a colored engraving of the microscopic appearance of the half of a lower molar, in which vessels charged with red blood are distinctly seen.

But if the vascularity of dentine never had been demonstrated, there are circumstances which would seem to establish the fact very conclusively. It is admitted, we believe, by all physiologists, that the solid materials of the body are derived from the red part of the blood. Now if this be true, how can we account for the increase of density which dentine acquires subsequently to its formation. This increase, it is true, is never very considerable, but that it does take place, is, we believe, very generally admitted by dentists. The manner in which it takes place, can only be explained by supposing that some of the red globules of the blood are broken down into such minute particles as to be capable of entering the delicate vessels of the dentine.

Having shown, as the writer believes, the two first assumptions of Dr. Neill to be erroneous, he will conclude with a very brief notice of his views concerning the supposed tubularity of dentine. Now, although this doctrine is held by several eminent men, who have devoted much time and attention to microscopical researches, Mr. Nasmyth has, we think, demonstrated in the most conclusive manner, that the supposed tubes are, in reality, "solid fibres," composed, to use his own language, "of a series of little masses, succeeding each other in a lineal direction, like so many beads collected on a string." Mr. N. has also demonstrated that both the fibres and interfibrous material of dentine have a cellular composition.

Upon this particular department of anatomy, Mr. Nasmyth

*Vide Maryland Med. and Surg. Jour., vol. ii, p. 385; Lectures on Dental Physiological Surgery, by John Tomes, pp. 49 and 50; and Principles and Practice of Dental Surgery, 4th edition, p. 47.

must be regarded as the highest authority—he having devoted some ten or twelve years of his life to the investigation of the minute structure of the teeth and their formative organs. But while we award to Mr. N. this credit, we would not be considered as detracting from the merits of Dr. Neill and other eminent gentlemen, who have been engaged in similar researches, and who, by their industry and splendid talents, have contributed so largely to the advancement of science.

Now, in view of the facts which the writer has thus hastily enumerated, he thinks it must be admitted by all, that the nerves of the dentine are the true media through which external impressions are transmitted, either directly to the nervous center, or to the pulp within, and from thence conveyed to it.

